# Imperatorin Restores Chemosensitivity of Multidrug-Resistant Cancer Cells by Antagonizing ABCG2-Mediated Drug Transport

**DOI:** 10.3390/ph16111595

**Published:** 2023-11-12

**Authors:** Chung-Pu Wu, Megumi Murakami, Yen-Ching Li, Yang-Hui Huang, Yu-Tzu Chang, Tai-Ho Hung, Yu-Shan Wu, Suresh V. Ambudkar

**Affiliations:** 1 Graduate Institute of Biomedical Sciences, College of Medicine, Chang Gung University, Taoyuan 33302, Taiwan; d1001301@cgu.edu.tw (Y.-C.L.); yanghui.huang01@gmail.com (Y.-H.H.);; 2 Department of Physiology and Pharmacology, College of Medicine, Chang Gung University, Taoyuan 33302, Taiwan; 3 Department of Obstetrics and Gynecology, Taipei Chang Gung Memorial Hospital, Taipei 10507, Taiwan; thh20@adm.cgmh.org.tw; 4 Laboratory of Cell Biology, Center for Cancer Research, National Cancer Institute, National Institutes of Health, Bethesda, MD 20892, USA; megumi.murakami@nih.gov; 5 Department of Medicine, College of Medicine, Chang Gung University, Taoyuan 33302, Taiwan; 6 Department of Obstetrics and Gynecology, Keelung Chang Gung Memorial Hospital, Keelung 20401, Taiwan; 7 Department of Chemistry, Tunghai University, Taichung 40704, Taiwan; yushanwu@thu.edu.tw

**Keywords:** ATP-binding cassette transporter, multidrug resistance, natural products, ABCG2, imperatorin

## Abstract

The high expression of the ATP-binding cassette (ABC) drug transporter ABCG2 in cancer cells contributes to the emergence of multidrug resistance (MDR) in individuals afflicted with either solid tumors or blood cancers. MDR poses a major impediment in the realm of clinical cancer chemotherapy. Recently, substantial endeavors have been dedicated to identifying bioactive compounds isolated from nature capable of counteracting ABCG2-mediated MDR in cancer cells. Imperatorin, a natural coumarin derivative renowned for its diverse pharmacological properties, has not previously been explored for its impact on cancer drug resistance. This study investigates the chemosensitizing potential of imperatorin in ABCG2-overexpressing cancer cells. Experimental results reveal that at sub-toxic concentrations, imperatorin significantly antagonizes the activity of ABCG2 and reverses ABCG2-mediated MDR in a concentration-dependent manner. Furthermore, biochemical data and in silico analysis of imperatorin docking to the inward-open conformation of human ABCG2 indicate that imperatorin directly interacts with multiple residues situated within the transmembrane substrate-binding pocket of ABCG2. Taken together, these results furnish substantiation that imperatorin holds promise for further evaluation as a potent inhibitor of ABCG2, warranting exploration in combination drug therapy to enhance the effectiveness of therapeutic agents for patients afflicted with tumors that exhibit high levels of ABCG2.

## 1. Introduction

An important challenge encountered in cancer chemotherapy is that, following extended treatment with conventional antiproliferative drugs, cancer cells can spontaneously lose their sensitivity to therapeutic agents that are structurally and mechanistically unrelated. This phenomenon is referred to as multidrug resistance (MDR) [1]. Among the various mechanisms contributing to the MDR phenotype in cancer, a significant factor is the high expression of ATP-binding cassette (ABC) drug transporters, particularly ABCB1 (P-glycoprotein/MDR1) and ABCG2 (BCRP/MXR/ABCP). Because these transporters could utilize energy obtained from ATP hydrolysis to actively expel a diverse array of chemicals, including a substantial number of therapeutic drugs [2,3], they are believed to play a central role in the development of MDR in cancer cells [3]. ABCB1 and ABCG2 are known to confer resistance to a large number of the most widely used conventional anticancer agents and molecularly targeted drugs [4,5,6].

Consequently, both transporters are associated with MDR and poor prognosis in patients with hematologic malignancies [7,8,9,10,11,12,13,14] and solid tumors [15,16]. Furthermore, heightened expression of ABCB1 and ABCG2 in cells comprising barrier sites between blood and tissues, such as the intestinal epithelium, the blood–brain barrier (BBB), and the blood–placenta barrier (BPB), can substantially impact the absorption, distribution, metabolism, elimination, and potential toxicity of a diverse range of therapeutic medications [2,3]. Considering that the persistence of MDR continues to present a significant challenge for drug developers and clinicians [2,3], the utilization of a targeted inhibitor to counteract the activity of ABCB1 or ABCG2 presents an appealing therapeutic avenue. The objective is to re-establish the sensitivity of multidrug-resistant cancer cells to anticancer medications [17]. However, the potential use of such inhibitors has been constrained by issues such as insufficient selectivity or unanticipated adverse effects arising from novel combination treatments [3]. Consequently, no agents have yet received approval from the U.S. Food and Drug Administration (FDA) for clinical application in combatting multidrug-resistant cancers. The exploration of utilizing bioactive compounds derived from plants, which are usually well-tolerated, to sensitize multidrug-resistant cancer cells has emerged as an alternative avenue [18,19]. A multitude of natural products have demonstrated the ability to reverse MDR in cancer cells that overexpress ABCB1 and ABCG2 [20]. Particularly noteworthy is the promising performance of several novel anticancer compounds derived from natural sources in both preclinical and clinical trials [21,22,23]. Imperatorin, a natural coumarin compound [24], can be isolated from various sources such as Notopterygium incisum (also referred to as *Hansenia weberbaueriana*) [25], *Rhodiola bupleuroides* [26], *Foeniculum vulgare* Mill [27], and *Angelica dahurica* [28]. Extensive research has detailed the pharmacological effects of imperatorin [29], encompassing its antibacterial [30], antiviral [31], antioxidant [32], antihypertensive [33,34], anti-inflammatory [35,36], hepatoprotective [37], and neuroprotective [38,39] properties. In addition, imperatorin has exhibited inhibitory effects on cell proliferation in a range of cancer types [40,41,42,43,44,45,46,47,48,49,50].

In this study, we focused on assessing the capability of imperatorin to reverse MDR conferred by ABCB1 and ABCG2. Our research revealed that imperatorin, when administered at sub-toxic concentrations, exhibits a distinct ability to selectively re-sensitize multidrug-resistant cancer cells that overexpress ABCG2 to antiproliferative drugs. Furthermore, we provide evidence that imperatorin achieves this reversal of ABCG2-mediated MDR by antagonizing the drug efflux function of ABCG2, all while leaving its protein expression level unaffected within these cancer cells. This conclusion gains additional support through the results of ATPase assays and molecular docking analyses. Collectively, our study underscores the potential of imperatorin as an adjunct to chemotherapy, augmenting the effectiveness of anticancer drugs.

## 2. Results

### 2.1. Imperatorin Reverses ABCG2-Mediated Multidrug Resistance in Cancer Cells

**Figure 1:**
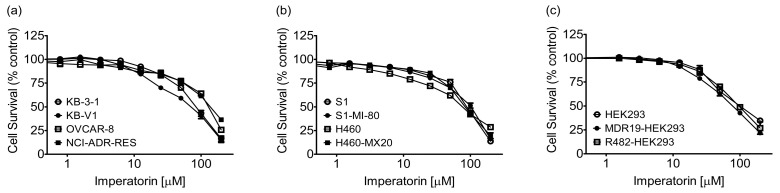
The intrinsic cytotoxic effects of imperatorin were examined in both drug-sensitive cell lines and their respective multidrug-resistant sublines that overexpress human ABCB1 or ABCG2. (**a**) Cell survival of the drug-sensitive KB-3-1 human epidermal cancer cell line (empty circles) and its ABCB1-overexpressing subline KB-V1 (filled circles) and of the drug-sensitive OVCAR-8 human ovarian cancer cell line (empty squares) and its ABCB1-overexpressing subline NCI-ADR-RES (filled squares). (**b**) Survival of the drug-sensitive S1 human colorectal cancer cell line (empty circles) and its ABCG2-overexpressing subline S1-MI-80 (filled circles) and of the drug-sensitive NCI-H460 cell line (empty squares) and its ABCG2-overexpressing subline H460-MX20 (filled squares). (**c**) Survival of human embryonic kidney 293 (HEK293) cells (empty circles), ABCB1-transfected HEK293 (MDR19-HEK293) cells (filled circles), and ABCG2-transfected HEK293 (R482-HEK293) cells (empty squares) after treatment with increasing concentrations of imperatorin for 72 h and processing as described in the Section 4. The data points indicate the mean values derived from a minimum of three independent experiments, with the error bars representing the SEM.

**Figure 2:**
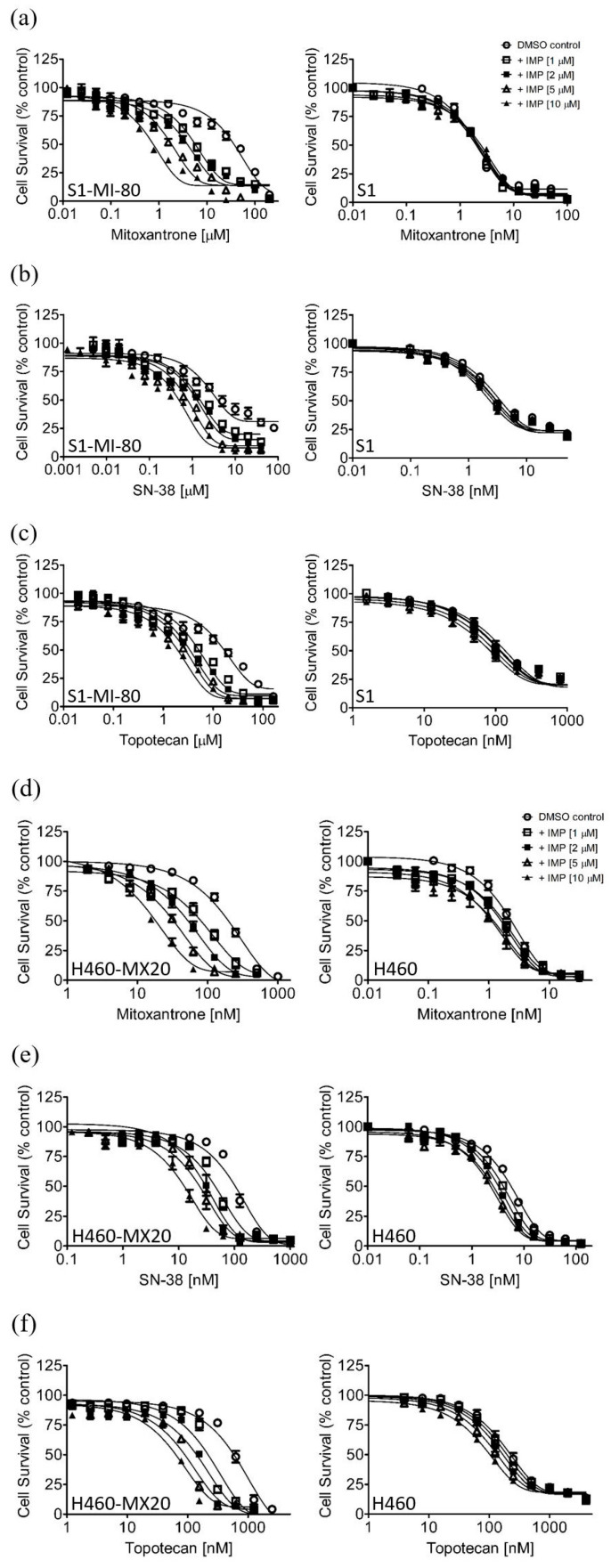

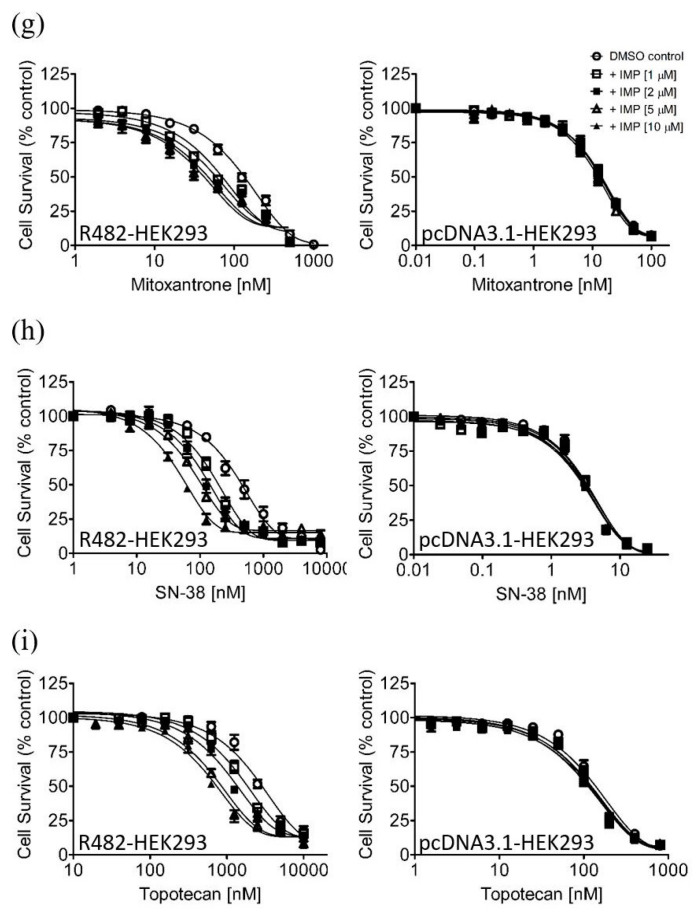
The chemosensitizing effect of imperatorin on ABCG2-overexpressing cells. The cytotoxicity of the ABCG2 substrate drugs mitoxantrone, SN-38, and topotecan in combination with the solvent control DMSO (open circles) or different concentrations of imperatorin (IMP) at 1 μM (open squares), 2 μM (filled squares), 5 μM (open triangles), or 10 μM (filled triangles), was determined in (**a**–**c**) S1-MI-80 (**left**) and S1 (**right**), (**d**–**f**) H460-MX20 (**left**) and H460 (**right**), and (**g**–**i**) R482-HEK293 (**left**) and pcDNA3.1-HEK293 (**right**) cells as described in the Section 4. The data points indicate the mean values derived from a minimum of three independent experiments, with the error bars representing the SEM.

Previous studies have reported that imperatorin possesses anti-proliferative activity against numerous cancer cell lines [40,41,42,43,44,45,46,47,48,49,50]. Consequently, we determined the cytotoxicity of imperatorin in the KB-3-1 and KB-V1 human epidermal cancer cell lines, the OVCAR-8 and NCI-ADR-RES human ovarian cancer cell lines, the S1 and S1-MI-80 human colon cancer cell lines, and the H460 and H460-MX20 human non-small-cell lung cancer (NSCLC) cell lines. As shown in Figure 1, imperatorin treatment significantly reduced the viability of cancer cells in a concentration-dependent manner. Crucially, imperatorin exhibited equivalent cytotoxicity towards both the drug-sensitive parental cell lines and the multidrug-resistant sublines that overexpress ABCB1 and ABCG2. The survival curves of the cells were analyzed to determine the ideal sub-toxic concentration of imperatorin, which was found to be 10 μM. This concentration was then used to evaluate the potential of imperatorin for sensitizing cells with ABCB1- and ABCG2-mediated MDR. We wish to point out that published work has demonstrated that imperatorin exhibits low general toxicity [29]. For instance, imperatorin demonstrated minimal toxicity, with over 80% cell viability observed at concentrations up to 100 µM in normal hippocampal neuronal cells [32] and up to 50 µM in mouse bone-marrow-derived mast cells [51]. In our present investigation, we assessed the cytotoxicity of imperatorin in human embryonic kidney (HEK293) cells, a widely employed mammalian expression system. The IC_50_ values for imperatorin in HEK293, ABCB1-transfected MDR19-HEK293, and ABCG2-transfected R482-HEK293 cells exceeded 70 µM (Figure 1C). Next, the impact of imperatorin on cytotoxicity was investigated in various cell lines. The cytotoxicity of three ABCB1 substrates, colchicine, paclitaxel, and vincristine [52], was assessed in KB-V1 and KB-3-1, NCI-ADR-RES and OVCAR-8, as well as MDR19-HEK293 and pcDNA3.1-HEK293 cell lines. Similarly, the cytotoxicity of three ABCG2 substrates [53,54,55], mitoxantrone, SN-38, and topotecan, was evaluated in S1-MI-80 and S1 (Figure 2a–c), H460-MX20 and H460 (Figure 2d–f), and R482-HEK293 and pcDNA3.1-HEK293 (Figure 2g–i) cell lines. These assessments were conducted in the presence or absence of imperatorin, allowing for the evaluation of imperatorin’s potential to modulate drug resistance. The results showed that imperatorin had no significant impact on the cytotoxicity of ABCB1 substrate drugs in cell lines overexpressing ABCB1. However, imperatorin significantly restored the sensitivity of cell lines overexpressing ABCG2 to ABCG2 substrate drugs in a concentration-dependent manner. These findings are summarized in Table 1 and Table 2. The extent of sensitization resulting from imperatorin in these multidrug-resistant cell lines was quantified using the fold reversal (FR) value [56]. The FR value is determined by dividing the IC_50_ value of a specific substrate drug by the IC_50_ value of the same substrate drug in the presence of imperatorin, acting as a modulator. It is noteworthy that, aside from the endogenous ABCG2-expressing H460 cell line [57], imperatorin did not markedly influence the cytotoxicity of substrate drugs in the parental cell lines. Moreover, the reference inhibitors tariquidar and Ko143 were employed as positive controls for restoring full sensitivity of ABCB1- and ABCG2-overexpressing multidrug-resistant cells to cytotoxic anticancer drugs.

### 2.2. Imperatorin Potentiates Topotecan-Induced Apoptosis in ABCG2-Overexpressing Cancer Cells

**Figure 3:**
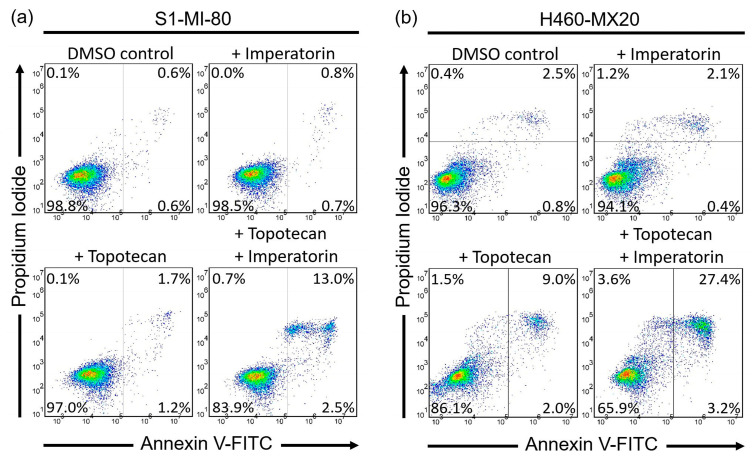

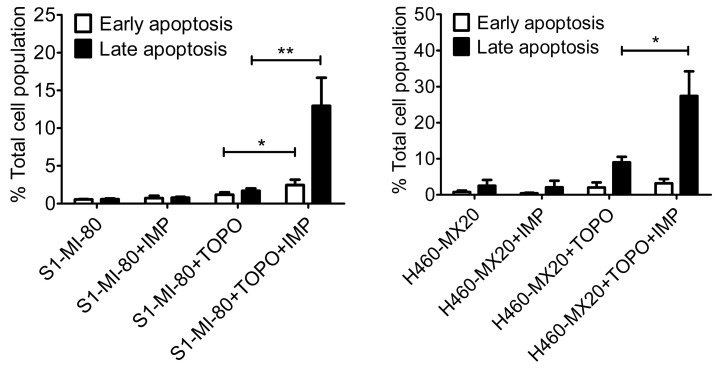
The effect of imperatorin on substrate drug-induced apoptosis in ABCG2-overexpressing multidrug-resistant cancer cells. (**a**) S1-MI-80 and (**b**) H460-MX20 cancer cells were treated with either DMSO (control), 10 μM imperatorin (+IMP), 10 μM topotecan (+ TOPO), or a combination of 10 μM topotecan and 10 μM imperatorin (+TOPO +IMP) for 48 h, as described in the Section 4. Following treatment, cells were subjected to the Annexin V-FITC and PI staining method and then assessed using flow cytometry analysis. The results are presented as representative dot plots (**left**) and quantified values (**right**), which were calculated as mean values ± SD from at least three independent experiments. Statistical significance was determined by a two-sided Student’s *t*-test and denoted as * *p* < 0.05; ** *p* < 0.01 when comparing the treatment group with imperatorin to the same treatment group without imperatorin.

In order to substantiate cytotoxicity and growth retardation caused by a drug substrate of ABCG2, the effect of imperatorin on apoptosis induced by topotecan was explored in S1-MI-80 and H460-MX20 cancer cells. The cells were treated with annexin V-FITC and stained with propidium iodide (PI) using the method outlined in the Section 4. S1-MI-80 (Figure 3a) and H460-MX20 (Figure 3b) cells were cultured with DMSO (control), 10 μM imperatorin, 10 μM topotecan, or a combination of 10 μM topotecan and 10 μM imperatorin for 48 h. Imperatorin notably amplified the apoptotic cell population induced by topotecan (increasing from 3% to 15% in S1-MI-80 and from 11% to 31% in H460-MX20). These results signify that imperatorin effectively enhances the apoptotic impact of the substrate drug of ABCG2 in multidrug-resistant cancer cells by reducing the activity of ABCG2.

### 2.3. Imperatorin Inhibits the Drug Efflux Function of ABCG2

**Figure 4:**
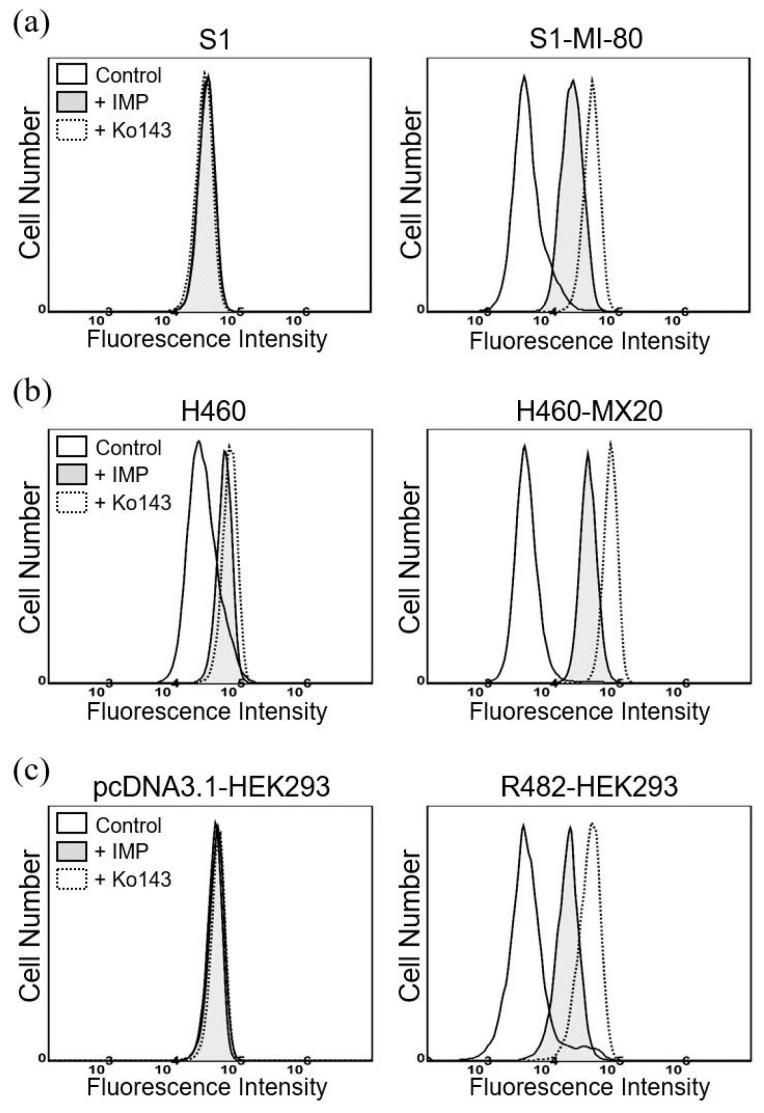
Imperatorin reduces the transport of fluorescent substrate mediated by ABCG2. The intracellular accumulation of the established fluorescent substrate, pheophorbide A (PhA), was assessed in (**a**) S1 and S1-MI-80, (**b**) H460 and H460-MX20, and (**c**) pcDNA3.1-HEK293 and R482-HEK293 cell lines. This assessment was conducted in the presence of DMSO (control, solid line), 10 μM imperatorin (+IMP, gray-shaded solid line), or 5 μM Ko143 (+Ko143, dotted line), which served as a positive control for ABCG2. The fluorescent signals were examined with flow cytometry, as outlined in the Section 4. The histograms depict representative outcomes from a minimum of three independent experiments.

Given that the direct inhibition of the efflux function of the drug transporter remains the most effective strategy for overcoming transporter-mediated MDR in cancer cells [18,58,59,60], we examined the effect of imperatorin on ABCG2-mediated efflux of an ABCG2-specific fluorescent substrate PhA [61] in S1-MI-80 and H460-MX20 cancer cells. This was accomplished by subjecting the cells to a 1 h incubation with PhA in the presence of DMSO (control), imperatorin (+IMP), or Ko143, following the methodology outlined in the Section 4. As shown in Figure 4, imperatorin at 10 μM notably inhibited the function of ABCG2 and restored the intracellular accumulation of PhA in ABCG2-overexpressing S1-MI-80 (Figure 4a) and H460-MX20 (Figure 4b) cancer cells, and ABCG2-transfected R482-HEK293 (Figure 4c) cells. The findings presented here are consistent with the outcomes of the MDR reversal assay, which demonstrate that imperatorin has the ability to reinstate the sensitivity of cancer cells that overexpress ABCG2 by enhancing the intracellular accumulation of ABCG2 substrate drugs. Of note, other than the endogenous ABCG2-expressing H460 cell line [57], imperatorin did not significantly increase the accumulation of any fluorescent probes in drug-sensitive parental cell lines (Figure 4a–c, left).

### 2.4. Imperatorin Does Not Induce Changes in the Protein Expression of ABCG2 in Multidrug-Resistant Cancer Cells

**Figure 5:**
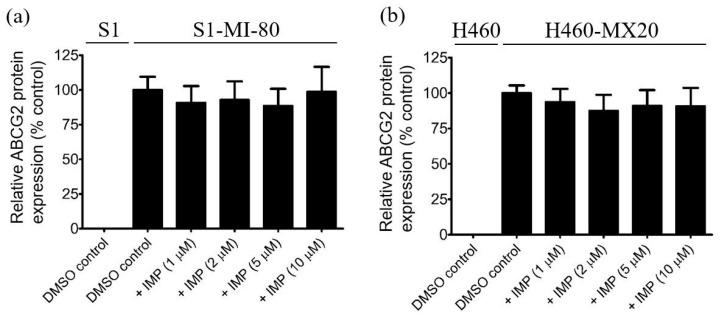
Imperatorin does not modify the protein expression of ABCG2 in cancer cells exhibiting multidrug resistance. ABCG2-overexpressing (**a**) S1-MI-80 and (**b**) H460-MX20 cancer cells were treated with DMSO (vehicle control) or imperatorin (IMP) at 1 μM, 2 μM, 5 μM, or 10 μM for 72 h. Subsequently, the cell lysates were subjected to immunoblot analysis, following the methodology outlined in the Section 4. The results were quantified and presented alongside α-tubulin levels, serving as the internal loading control. Values represent the mean ± standard deviation (SD) derived from at least three independent experiments. For statistical analysis, a two-sided Student’s *t*-test was employed. Original western blots are in Supplementary Materials.

Apart from directly inhibiting the drug efflux facilitated by a drug transporter, a prevalent mechanism through which multidrug-resistant cancer cells can regain sensitivity to drug treatment involves the drug-induced transient downregulation of the protein expression of the drug transporter [58,59,60]. For this purpose, the impact of imperatorin on the protein expression of ABCG2 was investigated in S1-MI-80 and H460-MX20 cancer cells. The cells were treated with escalating concentrations of imperatorin (1–10 μM) for a duration of 72 h, followed by immunoblotting (Figure S1) in accordance with the procedure outlined in the Section 4. In both the S1-MI-80 (Figure 5a) and H460-MX20 (Figure 5b) cancer cell lines, no notable alteration in the expression of ABCG2 was observed. This observation strongly suggests that imperatorin counters MDR in cells overexpressing ABCG2 by modulating the drug transport activity of ABCG2 rather than inducing changes in its protein expression.

### 2.5. Imperatorin Stimulates the ATPase Activity of ABCG2

**Figure 6:**
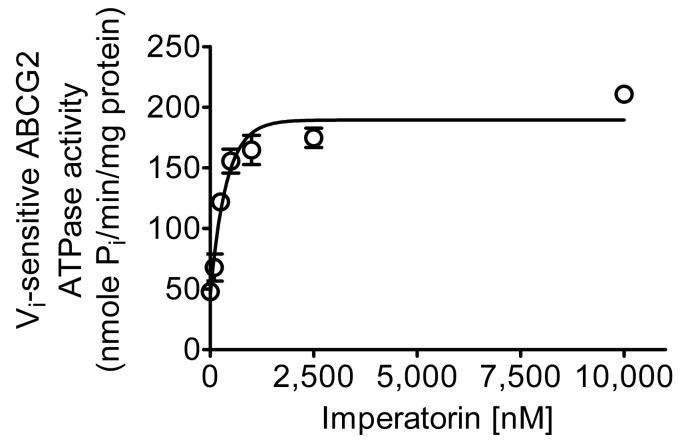
Imperatorin stimulates the vanadate (V_i_)-sensitive ATPase activity of ABCG2. To evaluate the effect of imperatorin on ABCG2-mediated ATP hydrolysis, imperatorin concentrations ranging from 0 to 10 μM were determined using endpoint P_i_ liberation assays. These assays utilized membrane vesicles derived from ABCG2 baculovirus-infected High Five insect cells, following a previously established protocol [68]. The data points represent the mean values derived from a minimum of three separate experiments, with error bars indicating the standard deviation (SD).

Given the established impact of substrates and inhibitors on ABCG2-mediated ATP hydrolysis [62,63,64,65], our investigation delved into the influence of imperatorin on the V_i_-sensitive ATPase activity of ABCG2. This analysis aimed to provide a deeper understanding of the interplay between imperatorin and ABCG2. As depicted in Figure 6, imperatorin elicited a concentration-dependent enhancement in the V_i_-sensitive ATPase activity of ABCG2. The maximal stimulation achieved was four-fold greater than the basal activity (measured at 47.87 ± 1.90 nmoles P_i_/min/mg protein), and the half-maximal effective concentration (EC_50_) was approximately 170 nM. These findings suggest that imperatorin binds and interacts with ABCG2 with relatively high affinity. This is in line with previous research that has shown the interaction between different chemosensitizing agents and the drug-binding pocket of ABCG2, leading to an increase in ABCG2-mediated ATP hydrolysis [56,66,67].

### 2.6. Docking of Imperatorin in the Drug-Binding Pocket of ABCG2

**Figure 7:**
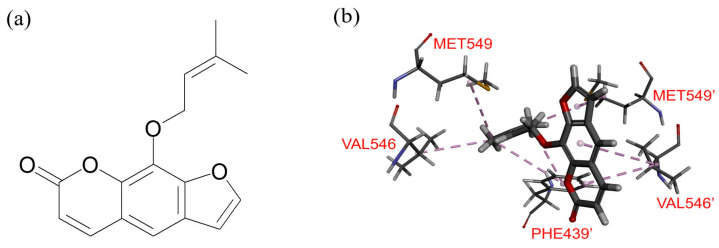
Common interactions observed between imperatorin and ABCG2 (**a**) Chemical structure of imperatorin. (**b**) Docking of imperatorin in the substrate-binding pocket of ABCG2. The lowest energy binding modes of imperatorin with ABCG2 protein structure (PDB:6VXH) were predicted by using BIOVIA Discovery Studio software version 4.0 as described in the Section 4. The molecular model of imperatorin and interacting amino acid residues are presented in stick representation and colored as follows: carbon—gray; hydrogen—light gray; nitrogen—blue; oxygen—red; and sulfur—yellow. Proposed interactions are presented as dotted lines.

To explore the specific amino acid residues interacting with imperatorin within the substrate-binding pocket of the human ABCG2 protein, a computational molecular docking analysis was performed. This analysis utilized the inward-open structure of ABCG2 retrieved from the Protein Data Bank (PDB: 6VXH), as detailed by Orlando and Liao in 2020 [69]. The results, as illustrated in Figure 7, showed several common interactions that are typically observed between substrates/inhibitors and the ABCG2 protein [69,70,71]. Notably, certain hydrophobic amino acid residues, Met549, Val546, Phe439′, Val546′, and Met549′, were predicted by the analysis to participate in *Pi–Pi/Pi*–alkyl interactions with the aromatic coumarin core structure of imperatorin.

## 3. Discussion

“Fourth-generation modulators” denote compounds that arise from natural origins, showcasing an extensive array of diversity and introducing novel chemical frameworks. Crucially, these compounds are often characterized by their minimal toxicity and heightened tolerance within the human body, making them particularly significant [19]. Interestingly, studies have reported that approximately 80% of cancer patients attempt to self-medicate by consuming natural products in addition to the prescribed anticancer medications, aiming to harness potential advantages in preventing chemotherapy-induced damage and obstructing the progression of cancer [72]. As a result, numerous research teams, our own included, have redirected their focus towards exploring the potential of employing bioactive compounds sourced from nature to counteract MDR facilitated by ABCB1 or ABCG2 [18,19,20,73]. In accordance with findings outlined in a recent review [20], various categories of natural products, ranging from alkaloids and flavonoids to carotenoids and terpenoids, exhibit the capability to modulate the activity of ABC drug transporters in human cancer cells. Nonetheless, it is important to recognize that not all natural product modulators of ABCG2 will be viable for clinical use. As an example, while the diketopiperazine fungal mycotoxin fumitremorgin C (FTC) stands out as one of the most potent ABCG2 inhibitors [74], its clinical applicability is curtailed by its neurotoxic properties [75].

Imperatorin, a widely recognized coumarin derivative, is readily available from diverse plant sources [25,26,27,28], and it has displayed anticancer activity against various types of cancer [40,41,42,43,44,45,46,47,48,49,50]. In the present investigation, we detailed the modulatory effects of imperatorin on ABCG2-mediated MDR in multidrug-resistant cancer cells. First, we evaluated the intrinsic cytotoxicity of imperatorin across both drug-sensitive parental cell lines and multidrug-resistant sublines in which ABCB1 or ABCG2 were overexpressed. Our findings indicated that imperatorin’s cytotoxicity remains consistent in both parental and multidrug-resistant cell lines, implying that it is not rapidly extruded from cancer cells by either ABCB1 or ABCG2 transporters (refer to Figure 1). Furthermore, we found that imperatorin, when administered at sub-toxic concentrations, substantially potentiated the cytotoxic effects of ABCG2 substrates such as mitoxantrone, SN-38, and topotecan in ABCG2-overexpressing cells (refer to Figure 2). In contrast, it had minimal impact on the cytotoxicity of ABCB1 substrates such as colchicine, paclitaxel, vincristine, and paclitaxel in ABCB1-overexpressing cells (refer to Table 1). It is worth noting that in a previous study, Liao and colleagues reported that imperatorin (1–10 µg/mL) exhibited the capability to restrain both the activity and gene expression of ABCB1 in MDCK-MDR1 (Madin–Darby canine kidney) cells, suggesting its potential to counteract MDR in tumors [76]. However, our findings reveal that imperatorin acts as a more potent modulator of ABCG2 in comparison to ABCB1, emphasizing its heightened specificity towards ABCG2.

To unravel the potential mechanism by which imperatorin sensitizes cancer cells overexpressing ABCG2, we conducted investigations into its impact on both the drug transport function and protein expression of ABCG2 in these cells. The results of our study indicate a strong inhibitory effect of imperatorin on ABCG2-mediated drug efflux, as evidenced in ABCG2-overexpressing cancer cells and HEK293 cells transfected with ABCG2 (R482-HEK293) (refer to Figure 4). Intriguingly, the overall protein level of ABCG2 in the ABCG2-overexpressing cancer cell lines remained largely unaffected following a 72 h incubation with imperatorin (refer to Figure 5). Additionally, our findings demonstrate a concentration-dependent stimulation of ABCG2 ATPase activity by imperatorin (as illustrated in Figure 6). The stimulation of ABCG2 ATPase activity is consistent with previous studies involving other competitive modulators of ABCG2, which also bind to the drug-binding pocket of ABCG2 and directly compete with transport substrates [56,64,77]. This effect was further supported by our molecular docking simulation, revealing several interactions between imperatorin and both monomers within the substrate binding cavity of ABCG2 (depicted in Figure 7b).

## 4. Materials and Methods

### 4.1. Materials and Cell Culture

Imperatorin was purchased from Selleckchem (Houston, TX, USA). Unless explicitly mentioned, all therapeutic compounds and other chemicals were sourced from Sigma-Aldrich (St. Louis, MO, USA). The TOOLS Cell Counting (CCK-8) kit was acquired from Biotools Co., Ltd. (Taipei, Taiwan). The Annexin V: FITC Apoptosis Detection Kit was purchased from BD Pharmingen (San Diego, CA, USA). The KB-3-1 and KB-V1 cell lines, the OVCAR-8 and NCI-ADR-RES cell lines, the H460 and H460-MX20 cell lines, pcDNA3.1-HEK293, human ABCB1-transfected MDR19-HEK293, and human ABCG2-transfected R482-HEK293 cells [78] were cultured in Dulbecco’s Modified Eagle’s Medium (Gibco Invitrogen, CA, USA). The S1 and S1-MI-80 cell lines were cultured in RPMI-1640 (Gibco Invitrogen, CA, USA). Multidrug-resistant cancer cell lines were maintained in medium containing respective drugs, as previously noted [78,79,80,81,82,83]. Cells were maintained in medium supplemented with 10% FCS, 2 mM L-glutamine, and 100 units of penicillin/streptomycin/mL (Gibco Invitrogen, CA, USA) at 37 °C in 5% CO_2_ humidified air and placed in drug-free medium 7 days prior to assay. Multidrug-resistant cell lines were generous gifts from Drs. Michael Gottesman and Susan Bates (NCI, NIH, Bethesda, MD, USA). The TOOLS Mycoplasma Detection Kit (Biotools Co., Ltd., Taipei, Taiwan) was routinely used to check for mycoplasma contamination.

### 4.2. Cell Viability Assay and Multidrug Resistance Reversal Assay

The sensitivity of cells to a specific agent was assessed using the CCK-8 and MTT assays, following the methodology outlined by Ishiyama et al. [84]. In brief, cells were seeded at a density of 5000 cells per well in 100 µL of culture medium into 96-well plates and incubated at 37 °C for 24 h before adding respective drugs or drug combinations, prepared in DMSO (up to 0.5% *v*/*v*), to achieve a final volume of 200 µL. Subsequently, cells were incubated for an additional 72 h with varying drug concentrations, followed by treatment with CCK-8 reagent or MTT, as previously described [85]. The IC_50_ (half-maximal inhibitory concentration) value for each treatment was computed from a fitted dose–response curve derived from a minimum of three independent experiments. For the reversal of cytotoxicity assays, a non-toxic concentration of imperatorin or tariquidar (a well-established ABCB1 inhibitor), or Ko143 (a well-established ABCG2 inhibitor), was added to the cytotoxicity assay. The extent of reversal was subsequently determined based on the relative resistance values and presented as the fold reversal (FR) value in accordance with previous descriptions [56].

### 4.3. Apoptosis Assay

To assess the impact of imperatorin on apoptosis induced by a cytotoxic drug substrate in multidrug-resistant cancer cells, apoptosis assays were conducted. The proportion of cells undergoing apoptosis within the overall cell population due to the specified treatment protocols was quantified using the conventional Annexin V–FITC and propidium iodide (PI) staining technique [86], as previously outlined [87]. In brief, cells were exposed to DMSO (control), 10 µM imperatorin, 10 µM topotecan, or a combination of topotecan and imperatorin, as indicated, for a duration of 48 h. Subsequently, the cells were harvested, centrifuged, and suspended in FACS buffer containing 1.25 µg/mL Annexin V–FITC and 0.1 mg/mL PI. Following a 15-min incubation at room temperature, the labeled cells (10,000 per sample) were subjected to analysis using FACScan equipment along with CellQuest software version 5.1 (BD Biosciences), employing the methodology elucidated previously [88].

### 4.4. Fluorescent Drug Accumulation Assay

The activity of ABCG2 was assessed by determining the intracellular accumulation of the fluorescent probe pheophorbide A (PhA), which emits light at 670 nm upon excitation at 395 nm. In brief, a cell count of 3 × 105 was harvested and suspended in 4 mL of IMDM supplemented with 5% FCS. PhA was then introduced into the cell suspension, along with imperatorin, tariquidar, or Ko143, either in the presence or absence of these compounds, and monitored for 1 h, as detailed in earlier works [61]. The fluorescence signal was captured using a FACScan flow cytometer from BD Biosciences and subjected to analysis using CellQuest software (Becton-Dickinson, Franklin Lakes, NJ, USA), along with FlowJo software version 7.6.1 (Tree Star, Inc., Ashland, OR, USA). The analytical approach employed was as outlined by Gribar et al. [89].

### 4.5. Immunoblotting

After incubating cells with varying concentrations of imperatorin for a duration of 72 h, the cells were collected and subsequently processed through SDS-polyacrylamide electrophoresis. For the Western blot immunoassay, primary antibodies BXP-21 (at a dilution of 1:10,000) and anti-alpha tubulin (at a dilution of 1:100,000) were employed to respectively target ABCG2 and the positive control tubulin. The secondary antibody used was horseradish peroxidase-conjugated goat anti-mouse IgG (at a dilution of 1:10,000). Detection and quantification of signals were carried out in accordance with previously established methods [67].

### 4.6. ATPase Assay

The effect of imperatorin on the vanadate (Vi)-sensitive ATPase activity of ABCG2 was assessed employing a colorimetric approach centered around an endpoint inorganic phosphate (Pi) assay, which quantifies the released Pi during the reaction, following established protocols [90].

### 4.7. Docking Analysis

The ABCG2 protein (PDB:6VXH) [69] and ligand preparation were performed using the CDOCKER module of Accelrys Discovery Studio 4.0. The energy minimization process for both the protein and imperatorin structures employed the CHARMM force field. For the subsequent docking analysis, the conformation of the ligand exhibiting the lowest CDOCKER interaction energy was selected in accordance with previously established methods [91].

### 4.8. Data Analysis

The experimental data and IC_50_ values are reported as mean ± standard deviation (SD) or mean ± standard error of the mean (SEM), derived from dose–response data collected in a minimum of three independent experiments unless specified otherwise. Curve fitting and statistical analysis were performed using GraphPad Prism version 5.0 (GraphPad Software, La Jolla, CA, USA) and KaleidaGraph version 5.0 (Synergy Software, Reading, PA, USA), respectively. The enhancement in fit was assessed using a two-sided Student’s *t*-test, and results were deemed “statistically significant” if the probability, *p*, was less than 0.05.

## 5. Conclusions

Collectively, our data strongly suggest that by competing with the binding of other substrate drugs within the substrate-binding pocket of ABCG2, imperatorin demonstrates selective reversal of ABCG2-mediated MDR. This competition effectively hampers the drug transport activity of ABCG2 (refer to Figure 7b). While it is important to acknowledge the potential involvement of other resistance mechanisms, the experimental findings presented here lend support to the idea that imperatorin counteracts ABCG2-mediated MDR through direct competition with the binding of concurrently administered chemotherapeutic drugs at the same site. Moreover, the likelihood of ABCG2 downregulation significantly contributing to this process seems limited. In summation, although the concomitant use of conventional anticancer agents and natural bioactive compounds might sometimes lead to unexpected drug–drug interactions, the experimental evidence provides a basis for considering imperatorin as a potential candidate for combination therapy in patients whose tumors exhibit elevated ABCG2 levels; this warrants further exploration through preclinical studies.

## Figures and Tables

**Table 1:** The effect of imperatorin on ABCB1-mediated drug resistance in ABCB1-overexpressing multidrug-resistant cell lines.

		Mean IC_50_^1^ ± SD and (FR ^2^)	
Treatment	Concentration (μM)	OVCAR-8 (Parental) [nM]	NCI-ADR-RES (Resistant) [μM]
Colchicine	-	34.08 ± 9.39 (1.0)	3.39 ± 0.53 (1.0)
+IMP	1	29.11 ± 8.90 (1.2)	4.23 ± 0.65 (0.8)
+IMP	2	40.97 ± 13.15 (0.8)	4.20 ± 0.67 (0.8)
+IMP	5	34.62 ± 9.70 (1.0)	4.73 ± 0.81 (0.7)
+IMP	10	33.81 ± 9.17 (1.0)	4.51 ± 0.69 (0.8)
+Tariquidar	1	28.85 ± 7.53 (1.2)	37.80 ± 12.70 [nM] *** (90)
		[nM]	[μM]
Vincristine	-	23.65 ± 2.88 (1.0)	5.77 ± 0.92 (1.0)
+IMP	1	26.40 ± 3.04 (0.9)	8.35 ± 1.72 (0.7)
+IMP	2	25.16 ± 2.55 (0.9)	8.63 ± 1.86 (0.7)
+IMP	5	24.11 ± 2.38 (1.0)	6.95 ± 1.56 (0.8)
+IMP	10	20.49 ± 2.75 (1.2)	5.23 ± 0.97 (1.1)
+Tariquidar	1	21.48 ± 2.32 (1.1)	76.34 ± 15.75 [nM] *** (76)
		[nM]	[μM]
Paclitaxel	-	4.67 ± 0.59 (1.0)	11.54 ± 2.05 (1.0)
+IMP	1	4.44 ± 0.55 (1.1)	11.10 ± 1.87 (1.0)
+IMP	2	4.92 ± 0.76 (0.9)	11.12 ± 2.12 (1.0)
+IMP	5	4.62 ± 0.55 (1.0)	12.35 ± 2.42 (0.9)
+IMP	10	4.23 ± 0.58 (1.1)	9.33 ± 1.22 (1.2)
+Tariquidar	1	4.82 ± 0.72 (1.0)	7.22 ± 0.73 [nM] *** (1598)
**Treatment**	**Concentration** **(μM)**	**KB-3-1** **(parental)** **[nM]**	**KB-V1** **(resistant)** **[μM]**
Colchicine	-	10.45 ± 3.44 (1.0)	1.38 ± 0.27 (1.0)
+IMP	1	9.74 ± 2.99 (1.1)	1.41 ± 0.17 (1.0)
+IMP	2	10.26 ± 3.50 (1.0)	1.53 ± 0.18 (0.9)
+IMP	5	11.34 ± 3.96 (0.9)	1.52 ± 0.17 (0.9)
+IMP	10	11.00 ± 3.83 (1.0)	2.01 ± 0.34 (0.7)
+Tariquidar	1	9.40 ± 2.97 (1.1)	12.52 ± 4.61 [nM] *** (110)
		[nM]	[μM]
Vincristine	-	5.00 ± 1.23 (1.0)	2.39 ± 0.43 (1.0)
+IMP	1	4.57 ± 1.08 (1.1)	2.36 ± 0.38 (1.0)
+IMP	2	4.38 ± 0.96 (1.1)	2.07 ± 0.36 (1.2)
+IMP	5	3.92 ± 0.92 (1.3)	1.84 ± 0.31 (1.3)
+IMP	10	3.64 ± 0.82 (1.4)	1.64 ± 0.30 (1.5)
+Tariquidar	1	3.74 ± 0.91 (1.3)	5.15 ± 1.06 [nM] (464)
		[nM]	[μM]
Paclitaxel	-	1.93 ± 0.68 (1.0)	3.54 ± 0.53 (1.0)
+IMP	1	1.95 ± 0.73 (1.0)	3.33 ± 0.50 (1.1)
+IMP	2	1.69 ± 0.47 (1.1)	3.46 ± 0.58 (1.0)
+IMP	5	1.81 ± 0.54 (1.1)	2.94 ± 0.50 (1.2)
+IMP	10	1.74 ± 0.50 (1.1)	2.70 ± 0.44 (1.3)
+Tariquidar	1	1.93 ± 0.70 (1.0)	1.83 ± 0.63 [nM] *** (1934)
**Treatment**	**Concentration** **(μM)**	**pcDNA3.1-HEK293** **(parental)** **[nM]**	**MDR19-HEK293** **(resistant)** **[nM]**
Colchicine	-	14.65 ± 4.29 (1.0)	313.26 ± 70.20 (1.0)
+IMP	1	13.49 ± 2.96 (1.1)	341.91 ± 63.09 (0.9)
+IMP	2	15.13 ± 3.87 (1.0)	408.47 ± 55.45 (0.8)
+IMP	5	15.01 ± 3.60 (1.0)	419.27 ± 63.99 (0.7)
+IMP	10	12.49 ± 3.58 (1.2)	427.51 ± 45.23 (0.7)
+Tariquidar	1	12.75 ± 3.90 (1.1)	13.06 ± 3.42 ** (24.0)
		[nM]	[μM]
Vincristine	-	17.14 ± 3.75 (1.0)	1.75 ± 0.23 (1.0)
+IMP	1	16.87 ± 3.24 (1.0)	1.93 ± 0.19 (0.9)
+IMP	2	17.89 ± 3.33 (1.0)	1.41 ± 0.22 (1.2)
+IMP	5	15.66 ± 2.98 (1.1)	1.54 ± 0.16 (1.1)
+IMP	10	14.88 ± 2.69 (1.2)	1.48 ± 0.16 (1.2)
+Tariquidar	1	13.35 ± 2.87 (1.3)	19.49 ± 2.13 [nM] *** (89.8)
		[nM]	[μM]
Paclitaxel	-	3.80 ± 0.87 (1.0)	4.76 ± 0.83 (1.0)
+IMP	1	3.10 ± 0.53 (1.2)	4.00 ± 0.62 (1.2)
+IMP	2	3.19 ± 0.58 (1.2)	3.30 ± 0.51 (1.4)
+IMP	5	3.47 ± 0.64 (1.1)	3.47 ± 0.49 (1.4)
+IMP	10	2.76 ± 0.48 (1.4)	3.39 ± 0.58 (1.4)
+Tariquidar	1	2.48 ± 0.41 (1.5)	4.76 ± 0.72 [nM] *** (1000)

Abbreviations: IMP, imperatorin; FR, fold reversal. ^1^ IC_50_ values are mean ± SD calculated from at least three independent experiments. ^2^ The FR value was determined by dividing the IC_50_ value of a specific substrate drug by the IC_50_ value of the same substrate drug in the presence of imperatorin or tariquidar. For statistical analysis, a two-sided Student’s *t*-test was employed. ** *p* < 0.01; *** *p* < 0.001.

**Table 2:** Imperatorin sensitizes ABCG2-overexpressing multidrug-resistant cells to cytotoxic drugs.

		Mean IC_50_^1^ ± SD and (FR ^2^)	
Treatment	Concentration (μM)	S1 (Parental) [nM]	S1-MI-80 (Resistant) [μM]
Mitoxantrone	-	1.97 ± 0.34 (1.0)	37.48 ± 5.45 (1.0)
+IMP	1	1.97 ± 0.26 (1.0)	7.02 ± 0.87 *** (5.3)
+IMP	2	2.07 ± 0.25 (1.0)	5.19 ± 0.84 *** (7.2)
+IMP	5	2.16 ± 0.23 (0.9)	2.11 ± 0.26 *** (17.8)
+IMP	10	2.39 ± 0.24 (0.8)	0.89 ± 0.10 *** (42.1)
+Ko143	1	1.91 ± 0.31 (1.0)	0.26 ± 0.04 *** (144.2)
		[nM]	[μM]
SN-38	-	5.38 ± 1.12 (1.0)	10.46 ± 2.71 (1.0)
+IMP	1	4.43 ± 0.86 (1.2)	2.21 ± 0.46 ** (4.7)
+IMP	2	4.35 ± 0.94 (1.2)	1.60 ± 0.33 ** (6.5)
+IMP	5	5.00 ± 1.17 (1.1)	797.97 ± 142.95 [nM] ** (13.1)
+IMP	10	3.91 ± 1.00 (1.4)	501.15 ± 91.75 [nM] ** (20.9)
+Ko143	1	4.34 ± 0.91 (1.2)	164.19 ± 33.28 [nM] ** (65.4)
		[nM]	[μM]
Topotecan	-	163.87 ± 19.28 (1.0)	19.83 ± 2.43 (1.0)
+IMP	1	153.34 ± 23.11 (1.1)	5.15 ± 0.67 *** (3.9)
+IMP	2	144.40 ± 22.21 (1.1)	3.35 ± 0.48 *** (5.9)
+IMP	5	135.88 ± 22.43 (1.2)	2.65 ± 0.27 *** (7.5)
+IMP	10	120.50 ± 21.51 (1.4)	1.93 ± 0.24 *** (10.3)
+Ko143	1	146.15 ± 18.37 (1.1)	1.00 ± 0.12 *** (19.8)
**Treatment**	**Concentration** **(μM)**	**H460** **(parental)** **[nM]**	**H460-MX20** **(resistant)** **[nM]**
Mitoxantrone	-	1.89 ± 0.34 (1.0)	168.24 ± 38.97 (1.0)
+IMP	1	1.68 ± 0.22 (1.1)	76.44 ± 13.14 * (2.2)
+IMP	2	1.46 ± 0.18 (1.3)	46.79 ± 7.12 ** (3.6)
+IMP	5	1.11 ± 0.13 * (1.7)	29.96 ± 5.22 ** (5.6)
+IMP	10	1.23 ± 0.20 * (1.5)	15.07 ± 2.72 ** (11.2)
+Ko143	1	1.35 ± 0.22 (1.4)	9.99 ± 1.46 ** (16.8)
		[nM]	[nM]
SN-38	-	8.36 ± 1.13 (1.0)	341.27 ± 104.72 (1.0)
+IMP	1	5.68 ± 0.65 * (1.5)	162.63 ± 57.39 (2.1)
+IMP	2	4.67 ± 0.67 ** (1.8)	113.89 ± 42.89 * (3.0)
+IMP	5	4.25 ± 0.75 ** (2.0)	89.98 ± 36.16 * (3.8)
+IMP	10	3.58 ± 0.69 ** (2.3)	75.92 ± 34.46 * (4.5)
+Ko143	1	2.29 ± 0.47 ** (3.7)	8.54 ± 2.92 ** (40.0)
		[nM]	[nM]
Topotecan	-	244.27 ± 27.29 (1.0)	1833.76 ± 465.26 (1.0)
+IMP	1	215.26 ± 26.33 (1.1)	700.29 ± 226.08 * (2.6)
+IMP	2	186.94 ± 23.76 (1.3)	618.64 ± 214.38 * (3.0)
+IMP	5	160.41 ± 23.62 * (1.5)	467.41 ± 169.84 ** (3.9)
+IMP	10	130.68 ± 25.03 ** (1.9)	333.46 ± 120.22 ** (5.5)
+Ko143	1	116.24 ± 17.19 ** (2.1)	88.12 ± 30.74 ** (20.8)
**Treatment**	**Concentration** **(μM)**	**pcDNA3.1-HEK293 (parental)** **[nM]**	**R482-HEK293** **(resistant)** **[nM]**
Mitoxantrone	-	13.36 ± 1.17 (1.0)	111.37 ± 14.83 (1.0)
+IMP	1	12.80 ± 1.25 (1.0)	62.41 ± 7.18 ** (1.8)
+IMP	2	13.34 ± 1.40 (1.0)	58.94 ± 7.72 ** (1.9)
+IMP	5	11.57 ± 1.33 (1.2)	48.56 ± 5.84 ** (2.3)
+IMP	10	12.58 ± 1.57 (1.1)	44.19 ± 5.01 ** (2..5)
+Ko143	1	12.24 ± 1.02 (1.1)	15.59 ± 1.30 *** (7.1)
		[nM]	[nM]
SN-38	-	3.02 ± 0.65 (1.0)	425.18 ± 27.55 (1.0)
+IMP	1	3.08 ± 0.67 (1.0)	180.40 ± 26.30 *** (2.4)
+IMP	2	2.77 ± 0.56 (1.1)	133.51 ± 19.25 *** (3.2)
+IMP	5	3.01 ± 0.64 (1.0)	110.63 ± 21.86 *** (3.8)
+IMP	10	2.82 ± 0.57 (1.1)	61.76 ± 13.87 *** (6.9)
+Ko143	1	2.61 ± 0.56 (1.2)	10.87 ± 1.62 *** (39.1)
		[nM]	[nM]
Topotecan	-	123.83 ± 25.91 (1.0)	2655.27 ± 495.12 (1.0)
+IMP	1	111.53 ± 20.56 (1.1)	1687.77 ± 271.40 * (1.6)
+IMP	2	105.94 ± 21.17 (1.2)	1275.03 ± 180.51 * (2.1)
+IMP	5	108.24 ± 20.17 (1.1)	844.84 ± 145.95 ** (3.1)
+IMP	10	104.79 ± 18.96 (1.2)	741.15 ± 103.20 ** (3.6)
+Ko143	1	107.26 ± 20.85 (1.2)	66.52 ± 17.79 *** (39.9)

Abbreviations: IMP, imperatorin; FR, fold reversal. ^1^ IC_50_ values are mean ± SD calculated from at least three independent experiments. ^2^ The FR value was determined by dividing the IC_50_ value of a specific substrate drug by the IC_50_ value of the same substrate drug in the presence of imperatorin or Ko143. For statistical analysis, a two-sided Student’s *t*-test was employed. * *p* < 0.05; ** *p* < 0.01; *** *p* < 0.001.

## Data Availability

Data are contained within the article.

## Supplementary Materials

The following supporting information can be downloaded at https://www.mdpi.com/article/10.3390/ph16111595/s1, Figure S1: original Western blots for Figure 5.
