# Supplementary figures and images for: Imperatorin Restores Chemosensitivity of Multidrug-Resistant Cancer Cells by Antagonizing ABCG2-Mediated Drug Transport
Source: Pharmaceuticals (Basel). 2023 Nov 12;16(11):1595. doi: 10.3390/ph16111595 (PMC10674403; doi:10.3390/ph16111595)

(a) S1-MI-80

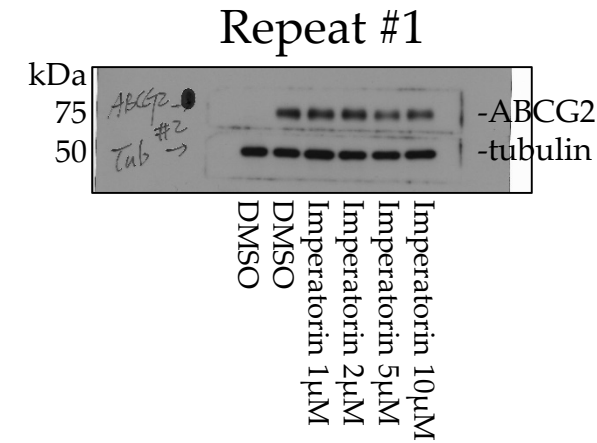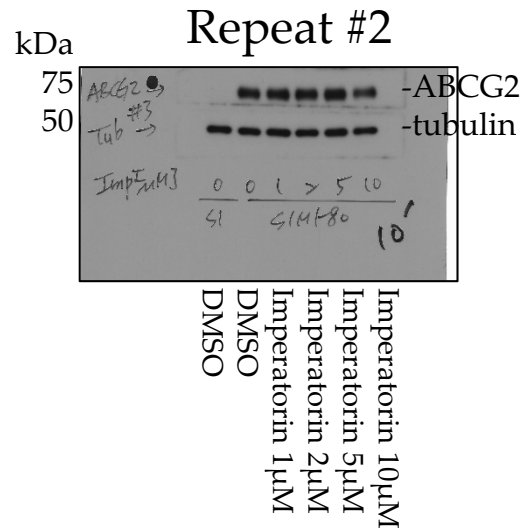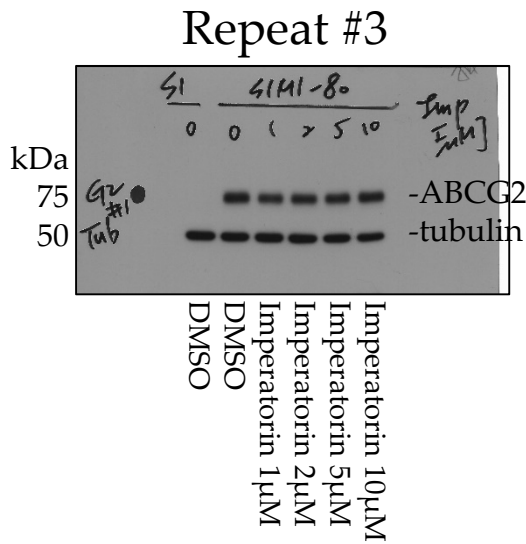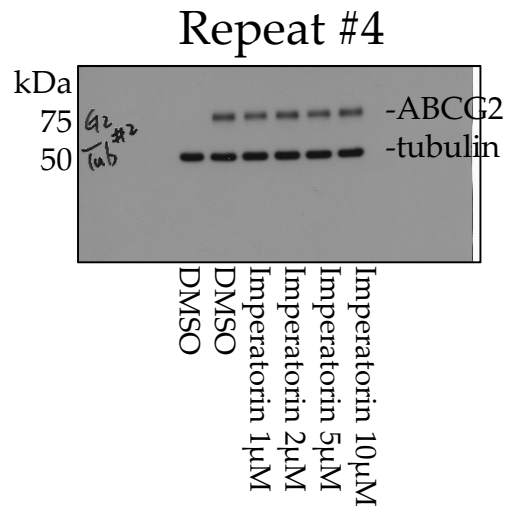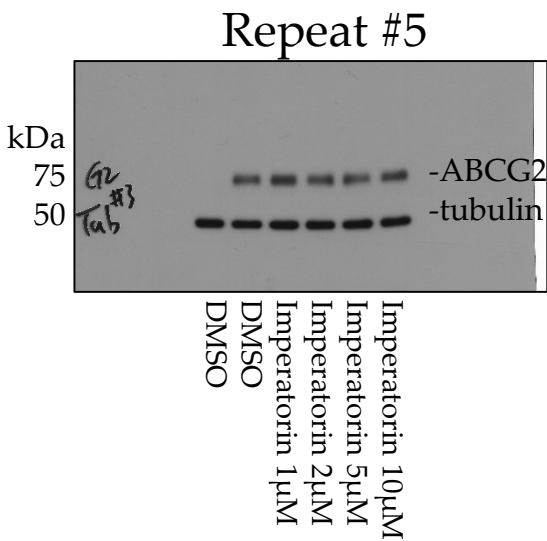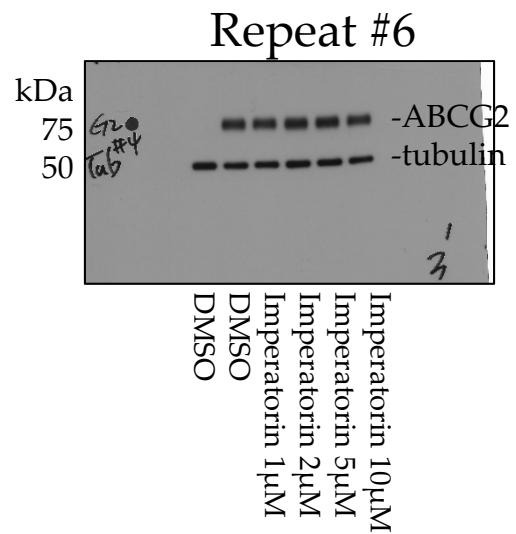

(b) H460-MX20

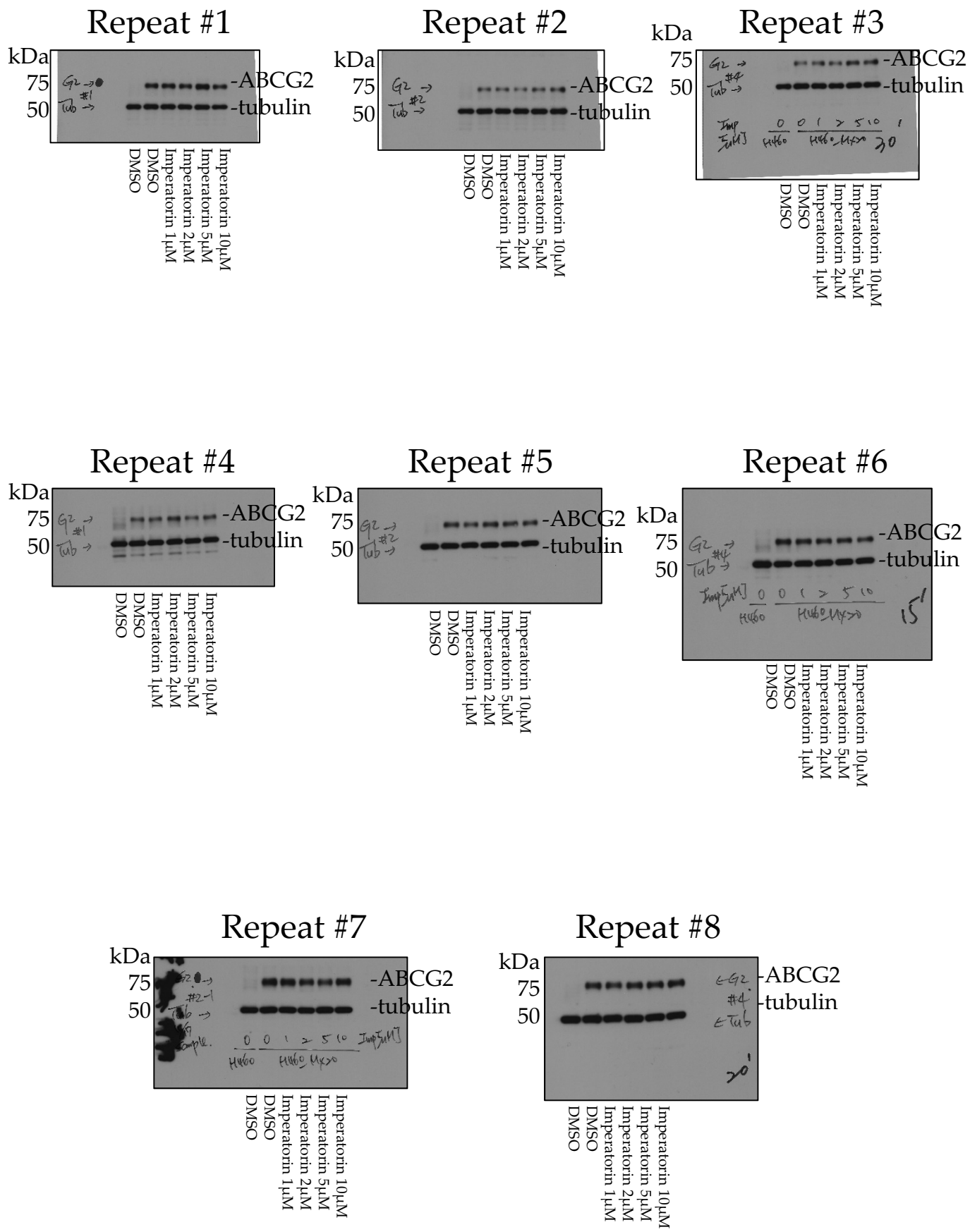

Figure S1: original Western blots for Figure 5.

Supplement: Supplementary file 1 [file pharmaceuticals-16-01595-s001.zip › pharmaceuticals-2669400-supplementary.pdf]
